# Short-term antidepressant treatment has long-lasting effects, and reverses stress-induced decreases in bone features in rats

**DOI:** 10.1038/s41398-018-0351-z

**Published:** 2019-01-16

**Authors:** S. H. Lee, C. A. Mastronardi, R. W. Li, G. Paz-Filho, E. G. Dutcher, M. D. Lewis, A. D. Vincent, P. N. Smith, S. R. Bornstein, J. Licinio, M. L. Wong

**Affiliations:** 1 John Curtin School of Medical Research, College of Health and Medicine, Australian National University, Canberra, ACT 0200 Australia; 2 Neuroscience Group (NeUROS), Institute of Translational Medicine, School of Medicine and Health Sciences, Universidad del Rosario, Bogotá, Colombia; 3 Trauma and Orthopaedic Research Laboratory, Department of Surgery, Medical School, Australian National University, Canberra, ACT 0200 Australia; 4 Mind & Brain Theme, South Australian Health and Medical Research, Adelaide, PO Box 11060, Adelaide, SA 5001 Australia; 5 School of Medicine, University of Adelaide, Adelaide, SA 5005 Australia; 6 Flinders University College of Medicine and Public Health, Bedford Park, SA 5042 Australia; 7 Freemasons Foundation Centre for Men’s Health, Department of Medicine, School of Medicine, University of Adelaide, Adelaide, SA 5005 Australia; 8 Clinical Orthopaedic Surgery, The Canberra Hospital, Yamba Drive, Garran, ACT 2605 Australia; 9 Medical Clinic III, Carl Gustav Carus University Hospital, Dresden University of Technology, Fetscherstraβe 74, 01307 Dresden, Germany; 10 Present Address: Section on Neural Gene Expression, National Institute of Mental Health, Building 49, Room 5A51, 49 Convent Drive, Bethesda, MD 20892 USA; 11 Present Address: Janssen Australia, 1-5 Khartoum Rd, North Ryde, NSW 2113 Australia; 12 Present Address: State of New York University, Upstate Medical University, Office of the Dean of Medicine, Room 1256 Weiskottem Hall, 766 Irving Ave, Syracuse, NY 13210 USA; 13 Present Address: State of New York, Upstate Medical University, 3738C NRB 505 Irving Ave, Syracuse, NY 13210 USA

## Abstract

Antidepressants are among the most-prescribed class of drugs in the world and though weight gain is a common outcome of antidepressant treatment, that effect is not well understood. We employed an animal model comprised of 2 weeks of chronic restraint stress with antidepressant treatment, followed by diet-induced obesity. We showed that short-term antidepressant treatment had long-lasting effects, not only leading to weight gain, but also enhancing trabecular and cortical bone features in rats; therefore, weight gain in this model was different from that of the classic diet-induced obesity. Late in the post-restraint recovery period, antidepressant-treated animals were significantly heavier and had better bone features than saline-treated controls, when assessed in the distal femoral metaphysis. The propensity to gain weight might have influenced the rate of catch-up growth and bone allometry, as heavier animals treated with fluoxetine also had enhanced bone features when compared to non-stressed animals. Therefore, short-term antidepressant treatment ameliorated the long-term effects of stress on body growth and bone. Growth and bone structural features were associated with leptin levels, and the interaction between leptin levels and antidepressant was significant for bone mineral content, suggesting that short-term antidepressants in the context of long-term diet-induced obesity modified the role of leptin in bone formation. To our knowledge this is the first study reporting that short-term antidepressant treatment has long-lasting effects in restoring the effects of chronic stress in body weight and bone formation. Our findings may be relevant to the understanding and treatment of osteoporosis, a condition of increasing prevalence due to the aging population.

## Introduction

Major depressive disorder (MDD) and obesity are both common heterogeneous disorders of complex etiology, and pronounced public health impact^1,2^. According to the data from the World Health Organization (WHO), MDD has become the second most prevalent cause of illness-induced disability, affecting 350 million people worldwide^3^. Concomitantly, obesity is a debilitating epidemic, affecting 36.5% of US adults^4^. Currently, given the high prevalence of obesity and mood disorders, it is conceivable that nearly 25% of the cases of obesity may be attributable to the association with MDD^5^. Cross-sectional and longitudinal studies have been conducted in order to understand the casual relationship between MDD and obesity^6–8^. Both disorders have in common the dysregulation of the hypothalamic–pituitary–adrenal axis, which is persistently activated during chronic stress^9^.

In the USA, antidepressant drugs were the second most prescribed class of drugs in 2011–2014^4^. Weight gain is a common outcome of antidepressant treatment. The interplay between MDD, obesity, and antidepressant-induced weight gain is complex. Though acute selective serotonin reuptake inhibitor (SSRI) treatment leads to weight loss, chronic SSRI treatment may lead to weight gain^10–12^. The discontinuation rate for antidepressant treatment is high within 2 months of treatment initiation, ranging from 70% for fluvoxamine, to 45% for fluoxetine (FX) and 40% for sertraline;^13–15^ thus, the lifetime prevalence of antidepressant exposure is very high. Based on the model that we have described previously^16^, here we combined 2-weeks of recurrent restraint stress and antidepressant treatment, followed by long-term diet-induced obesity; and referred it as the stress-antidepressant and diet-induced obesity (SADIO) model.

We hypothesized that increased body weight related to antidepressant treatment in the SADIO model had different pathophysiological mechanisms from those of the diet-induced obesity model. In the SADIO model described in this paper, we show that previously described body changes in the post-stress acclimation and recovery period^17^ included increased bone length, weight and structural changes. Furthermore, there was a signficant association between leptin and bone mineral content (BMC) in the SADIO model, which was not present in animals not exposed to antidepressants.

## Methods and materials

### Animals

Male Sprague-Dawley rats (170–190 g, 5–6 weeks old, Animal Resources Centre, Murdoch, WA, Australia) were housed one per cage at 24 °C and with a 12-h light/dark schedule (07:00 h to 19:00 h) in a stress-free environment. All the animal experimental procedures conducted were approved under protocol number J.MB.50.10, Animal Experimentation Ethics Committee, The Australian National University, Australia.

### SADIO animal paradigm

Animals were randomly allocated into four groups: three received chronic restraint-stress (CRS) and one group did not receive CRS (NR group, *N* = 30). The NR group did not receive CRS or intraperitoneally (i.p.) injections, but received the same dietary schedule as the CRS groups. For overview of the experimental protocol see supplementary Figure S1. Prior to CRS, there was no significant difference in body weight between the groups. CRS was applied from experimental days 5 to 19 for 6 h (from 9:00 to 15:00 h) using flat-bottom clear acrylic restrainers (Cat no. 544-RR Plas Labs, Lansing, MI, USA). During the CRS period, CRS groups received daily treatment with imipramine [IM, *N* = 13, 10 mg/kg i.p.; Sigma-Aldrich, St Louis, MO, USA], fluoxetine (FX, *N* = 14, 10 mg/kg ip; Eli Lilly, Indianapolis, Indiana, USA) or saline solution (CS, *N* = 10, 0.9% sodium chloride solution ip, Phebra, Lane Cove West, NSW, Australia) immediately prior to the CRS procedure. Imipramine was prepared in 24.5 mg/mL in 0.9% sodium chloride solution by mild vortexing and pH was adjusted to 7.4. The solution was further diluted and the final concentration of the imipramine was 4.9 mg/mL in 0.9% sodium chloride solution. Fluoxetine hydrochloride was prepared 5.6 mg/mL in 0.9% sodium chloride due to its low solubility and pH was adjusted to 7.4. The solution was further diluted to final concentration of 4.9 mg/mL in 0.9% sodium chloride solution. Solutions were filtered with a 0.22-μm filter (EMD Millipore™SLGP033RS, Ontario, Canada). After the CRS period, all groups of animals received a high-fat diet (18.6% fat semi-pure rodent diet SF10-020, Specialty Feeds, Glen Forrest, WA, Australia) to induce obesity from day 19 to 296 (276 days). Researcher could not be blinded to the experimental groups as daily i.p. injection of specific treatment group was necessary from days 5 to 19. We did not conduct power analysis prior to this study as there was no study using our animal model for 2 weeks of CRS followed by induced obesity from day 19 to 296. Body weight and food intake were measured daily during the CRS period and twice weekly thereafter. Due to the fact that rodents, such as Sprague-Dawley rats, display variable weight gain when fed a high-fat diet, animals were classified into subgroups of animals that gain significant weight (obesity prone) or not (obesity resistant)^18^. Therefore, we conducted analyses to understand whether the ability to gain weight during diet-induced obesity had significant effects in our model. Within each group, rats in the upper 50% of body weights were classified as the obesity-prone subgroup based on their body weight at the end of the experiment, and animals in the lower 50% were classified as the obesity-resistant subgroup.

#### Behavioral testing

Open-field tests and elevated plus maze were conducted during the post-stress acclimation period (experimental weeks 3–12), equipped with a camera tracking system (Viewer II system, Biobserve GmbH, Bonn, Germany). *Open-field test*: Trials were conducted for 30 min/sessions from 11:00 to 16:00 h. Rats were placed in the center of the field (48.8 cm × 48.8 cm × 50 cm). Total distance (TD), center distance (CD), and center distance to total distance (CD/TD) ratio were obtained and used as an index of anxiety^16^. Groups were CRS treated with saline, N = 11; FX, CRS treated with fluxotine, N = 14, and IM, CRS treated with imipramine, N = 11. Their means were subsequently averaged along the 10 sessions. *Elevated plus maze (EPM):* During the post-stress acclimation period (day 257), EPM test was performed to measure the level of anxiety-like behavior. EPM was elevated from the floor with two open arms and two closed arms (50 cm × 13 cm). Rats were placed in the middle of the maze facing a closed arm; trials were conducted for 5 min/session. The number of entries into the open arms was counted and the percentage of time spent in the open arms (ratio of open arms time/closed arms time) was calculated. The groups were CS, *N* = 11; FX, *N* = 12; IM, *N* = 11. Researchers were blinded while conducting behavioral tests.

### Dual energy X-ray absorptiometry (DXA) for body composition analysis and body length measurements

Body composition analysis of subsets of obesity-prone (upper 30%) and obesity-resistant (lower 30%) animals were obtained under anesthesia using the GE Lunar PIXImus equipment (Madison, WI, USA) and the Lunar imaging software (ver.1.46) according to standard procedures (see supplementary methods for detailed methodology). The groups were CS, *N* = 4; FX, *N* = 5; IM, *N* = 4; NR, *N* = 10.

### Microtomography (Micro-CT)

Micro-CT imaging of hind femurs was obtained from all animals using the Skyscan micro-CT equipment (Bruker, Kontich, Belgium). Distal femora were scanned submerged in 70% ethanol at a resolution of 21.3 μm. Transverse plane images were obtained from reconstructed images using the DataViewer software (Skyscan), and distal metaphyseal volume and mid-diaphyseal cortical geometry were generated by a binarized image program (CtAnalyzer software, Skyscan) (see supplementary methods for detailed methodology). The groups were CS, *N* = 10; FX, *N* = 14; and IM, *N* = 13, groups. NR, *N* = 28.

### Quantitative real time PCR (qPCR)

cDNA samples were tested in triplicate for the following rat genes: rat *Igf1* in liver tissue, groups were CS, *N* = 9; FX, *N* = 12; IM, *N* = 13; NR, *N* = 25; and 8 target genes in adipose tissue: *Tnf*, *Slc2a4*, *Pparg, Adipoq, Fasn, Lpl*, *Lipe*, and *Ppargc1a* (see supplementary methods for detailed methodology and primer sequences), groups were NR, *N* = 11; IM, *N* = 11; FX, *N* = 12; and CS, *N* = 8.

### Immunoassays

Commercial immunoassay kits were used to determine plasma IGF-1(CS, *N* = 7; FX, *N* = 8; IM, *N* = 8; NR, *N* = 16), leptin (CS, *N* = 10; FX, *N* = 14; IM, *N* = 13; NR *N* = 30), triglyceride (CS, *N* = 10; FX, *N* = 14; IM, *N* = 13; NR, *N* = 27), total cholesterol (CS, *N* = 10; FX, *N* = 14; IM, *N* = 13; NR, *N* = 30), free fatty acid (CS, *N* = 10; FX, *N* = 14; IM, *N* = 13; NR, *N* = 30), vanillylamandelic acid (CS, *N* = 10; FX, *N* = 14; IM, *N* = 13; NR, *N* = 26), and pituitary GH (CS, *N* = 6; FX, *N* = 7; IM, *N* = 6; NR, *N* = 10), following the manufacturers’ protocols (see supplementary methods for detailed methodology).

### Statistical analysis

Piecewise non-linear mixed effects growth curve models were constructed to estimate rat weights, *y*, post-stress over two periods: (A) days 20–60 and (B) day 60 onward. Analyses performed in R v3.4.1 (Vienna, Austria) using the *nlme* package (see supplementary methods for detailed statistical analysis)^19^.

Group comparisons were done by one-way analysis of variance (ANOVA) or non-parametric test of Kruskal-Wallis when the variances were not equal among treatment groups, and respectively followed by Tukey’s or Dunn’s post hoc test, using the GraphPad Prism 5.0 software (La Jolla, CA, USA) (see supplementary methods for detailed statistical analysis).

Associations between the five body composition outcomes [bone mineral content (BMC), bone mirenal density (BMD), % fat, body length, body weight) with four biochemical measures (log-transformed plasma leptin, total cholesterol, triglyceride, and fatty acids levels) were tested using linear regressions (see supplementary methods for detailed statistical analysis).

## Results

### Two-week antidepressant treatment improves body weight recovery during the post-stress period

**Fig. 1:**
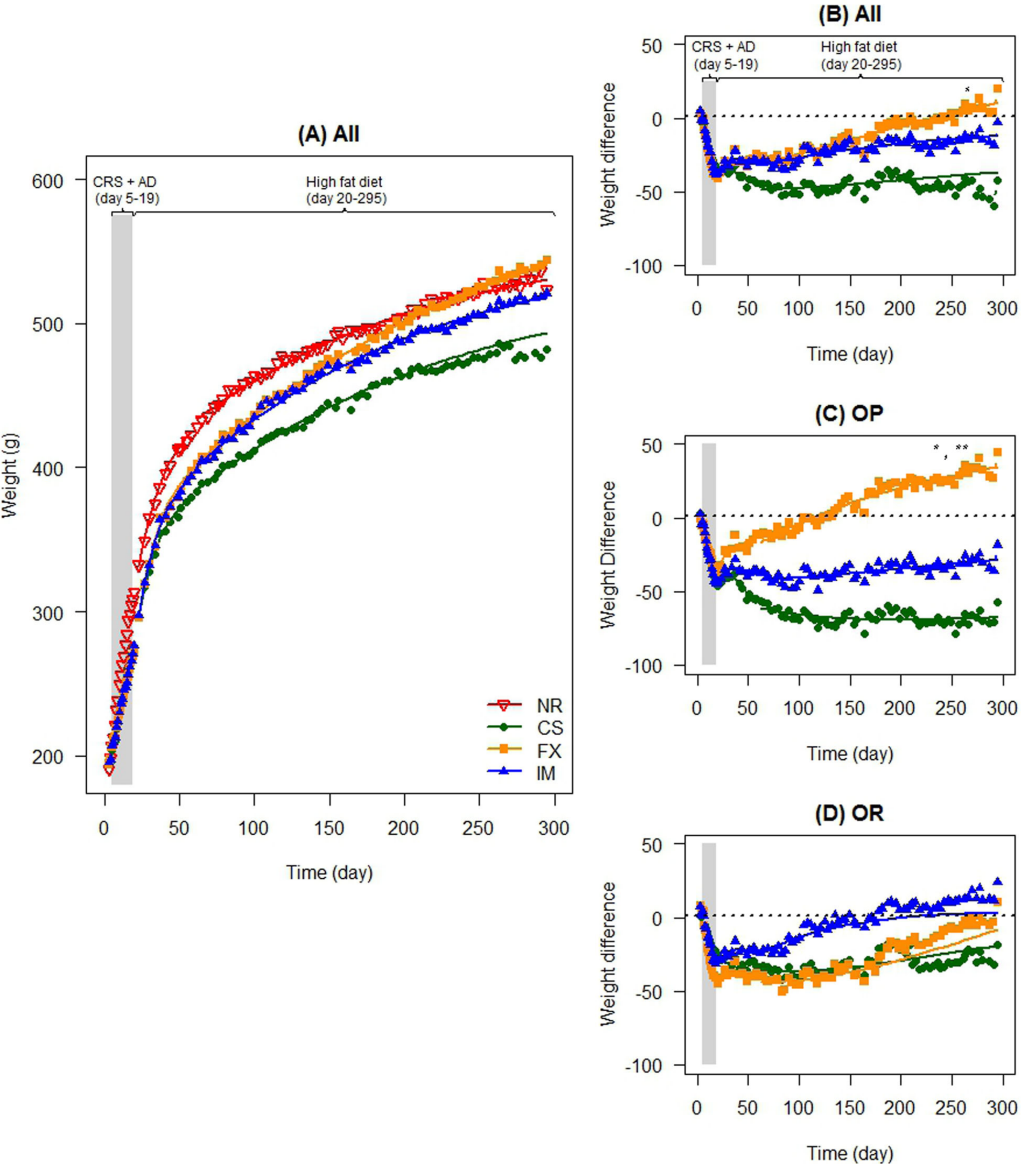
The effect of chronic restraint stress (CRS) and antidepressant on long-term weight gain and effect on classified subgroups of obesity prone (OP) and obesity resistant (OR) groups **a** Body weight for all groups during experimental days 3 to 296. CRS occurred in the CS (control CRS treated with saline, *N* = 10), FX (CRS treated with fluxotine, *N* = 14), and IM (CRS treated with imipramine, *N* = 13) groups between experimental days 5–19 (grey shaded area, CRS + antidepressant treatment, AD), followed by a high-fat diet from experimental days 19–296. **b**–**d** The graph represents the comparison of the difference in body weight [*body weight of* *antidepressant-treated group (FX, N* = *14 and IM, N* = *13)*—*non-restraint reference group (NR, N* = *30)*] in reference to difference in body weight of the restraint saline-treated control group [*body weight of saline-treated control group (CS, N* = *10)—non-restraint reference group (NR* = *30)*]. CRS occurred in the CS, FX, and IM groups between experimental days 5–19 (grey shaded area), followed by a high-fat diet from experimental days 19–296. (B) Difference in body weight of all groups, **c** difference in body weight of OP subgroups, (D) difference in body weight of OR subgroups. *P < 0.05, **P < 0.01, ***P < 0.001

Immediately post CRS, groups treated with FX, imipramine (IM) or vehicle (CS) had significantly lower body weight (${{\hat \beta} _0^{1}}$ all *P* < 0.001) than the non-stressed reference (NR) group (SI Appendix, Table S1A). During the post CRS period, the group treated with FX gained more body weight than the saline control (CS) group (Table S1 B, *P* = 0.03). Furthermore, the FX group became heavier than the NR group (Fig. 1a, b). We did not detect differences in the weights of the IM and CS-treated rats (*P* = 0.10).

### Chronic stressed obesity prone and obesity resistant subgroups treated with antidepressant had better weight recovery in the post-acclimation period

The log-likelihood ratio tests indicated the presence of interactions between treatment and obesity-prone groups in both periods (both *P* < 0.0001; SI Appendix, Table S1 BC). In the obesity-prone (upper 50% body weight) subgroups, the FX-treated group gained more body weight than the other CRS-treated groups (obesity prone: CS vs FX *P* < 0.0001, IM vs FX *P* = 0.01; Fig. 1c, and SI Appendix Table S1 B). In contrast, amongst the obesity-resistant (lower 50% body weight) stressed subgroups, body weights did not differ (Fig. 1d, and SI Appendix Table S1 B).

### Chronic stressed FX-treated animals were less anxious

**Fig. 2:**
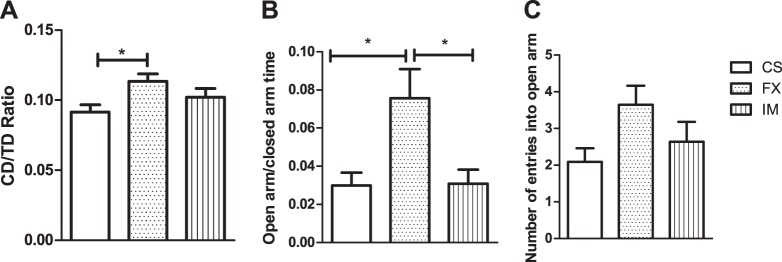
Interaction between exposure to chronic restraint stress (CRS), short-term antidepressant treatment and high-fat diet, and effects on anxiety and locomotor activity during the post-restraint stress period **a** Locomotor activity in the open field. (CD/TD) ratio in the openfield box during 30 min of locomotor activity testing; CS, CRS treated with saline, *N* = 11; FX, CRS treated with fluxotine, *N* = 14, and IM, CRS treated with imipramine, *N* = 11. **b**, **c** Elevated plus maze. **b** Time spent in open arm to closed arm ratio. **c** Number of entries into open arm. CS, *N* = 11; FX, *N* = 12; IM, *N* = 11. Results are shown as means ± s.e.m. *P < 0.05

During the post-restraint stress period (weeks 3–12), we conducted behavioral tests on animals that had undergone CRS. The FX group was significantly less anxious than the CS group, based on the open field test measure of CD/TD ratio, and on the open/closed arm ratio on the elevated plus maze test (both *P* < 0.05, Fig. 2a, b, and SI Appendix, Table S2).

### Bone morphological features were enhanced in chronic stressed antidepressant-treated animals

**Fig. 3:**
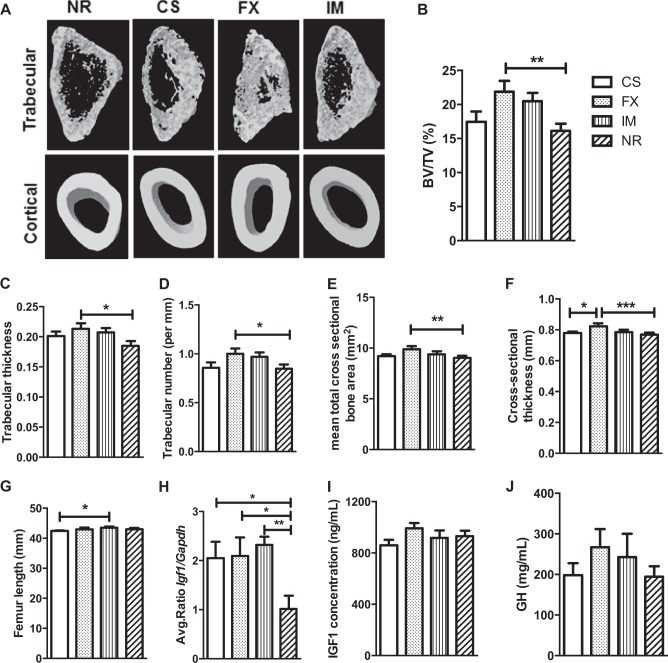
Effect of chronic restraint stress (CRS) and antidepressants on femoral bone morphology, and insulin-like growth factor 1 (IGF1) and growth hormone (GH) measurements during post-stress acclimation period **a**–**d** Trabecular bone analysis: **a** micro-computed tomography (CT) images of trabecular and cortical bones, **b** trabecular bone volume/total volume %, BV/TV %, **c** trabecular thickness, **d** trabecular number. **e**, **f** Cortical bone analysis: **e** mean total cross sectional bone area and **f** bone cross sectional thickness obtained from micro-CT analysis. CS, CRS treated with saline, *N* = 10; FX, CRS treated with flouxetine, *N* = 14, and IM CRS treated with imipramine, *N* = 13, groups. NR, non-CRS reference group, *N* = 28. **g** Femur length CS (*n* = 10), FX (*n* = 14), IM (*n* = 13), NR (*n* = 29). **h** Comparison of mean Igf1/Gapdh gene expression between treatments, CS, *N* = 9; FX, *N* = 12; IM, *N* = 13; NR, *N* = 25. **i** IGF1 plasma concentration (ng/mL), CS, *N* = 7; FX, *N* = 8; IM, *N* = 8; NR, *N* = 16. **j** GH pituitary concentration (mg/mL), CS, *N* = 6; FX, *N* = 7; IM, *N* = 6; NR, *N* = 10. Results are shown as means ± s.e.m. *P < 0.05, **P < 0.01, ***P < 0.001

Our findings support that the FX group had significantly enhanced trabecular and cortical bone micro-CT features (Fig. 3a). Percentage of trabecular bone volume/total volume ratio (BV/TV %), and trabecular thickness and number were significantly greater in the FX group in comparison to the NR group (respectively, *P* < 0.01, *P* < 0.05, and *P* < 0.05, Fig. 3a–d, and SI Appendix, Table S3). Cortical bone features, such as mean total cross-sectional bone area and thickness, were also significantly greater in the FX group in comparison to the NR group (respectively, *P* < 0.01, *P* < 0.001, and Fig. 3e, f, and SI Appendix, Table S3). The FX group also had mean total cross-sectional thickness significantly greater than the CS groups (*P* < 0.05, Fig. 3f, and SI Appendix, Table S3). The femur length was significantly greater in the IM in comparison to the CS group (*P* < 0.05, Fig. 3g, and SI Appendix, Table S3).

### Hepatic *Igf1 (insulin growth factor 1)* mRNA levels were increased in chronic stressed animals

At the end of the experiment, hepatic *Igf1* gene expression was significantly higher in all CRS groups in comparison to the NR group (*P* < 0.05 for CS and FX, and *P* < 0.01 for IM, Fig. 3h, and SI Appendix, Table S2). However, IGF-1 and growth hormone (GH) plasma levels were not significantly different between the groups (respectively, Fig. 3I, j, and SI Appendix, Table S2).

### Chronic stressed obesity prone saline-treated animals had lower body weight and accumulated food intake, and higher food intake ratio

**Fig. 4:**
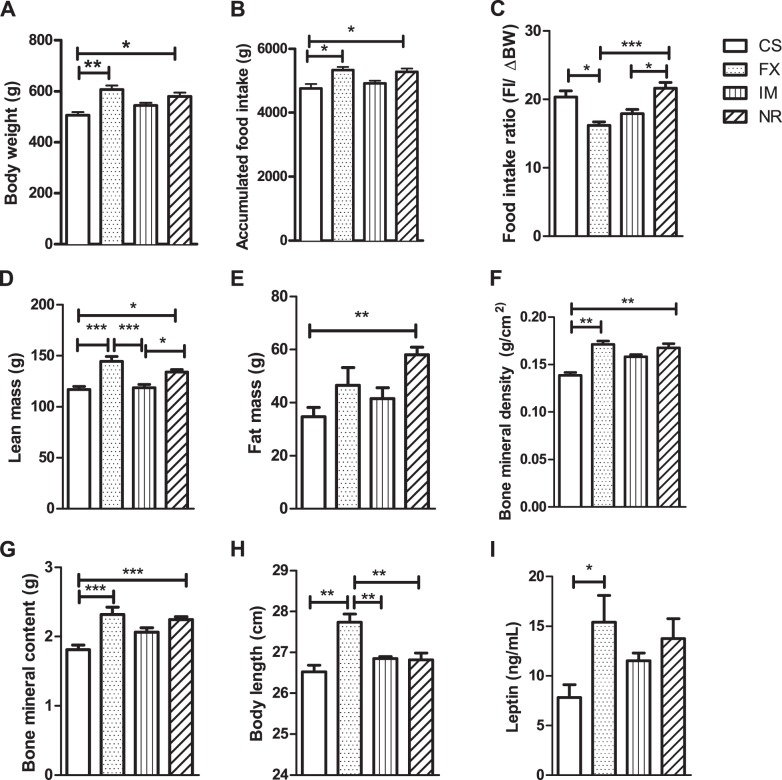
The effect of chronic restraint stress (CRS) and antidepressant on body weight and composition during post-stress acclimation period **a** Body weight of OP animals on day 296. **b** Accumulated food intake in obesity prone animals. CS, CRS treated with saline *N* = 5; FX, CRS treated with fluxotine, *N* = 7, and IM, CRS treated with imipramine, *N* = 7, groups. NR, non-CRS reference group, *N* = 15. **c**–**g** DXA (Dual energy x-ray absorptiometry scan) data for OP animals prior to euthanasia. **c** Lean mass, **d** fat mass, **e** bone mineral density, **f** bone mineral content, and **g** body length, CS, *N* = 4; FX, *N* = 5; IM, *N* = 4; NR, *N* = 10. H Femur length, CS, *N* = 10; FX, *N* = 14; IM, *N* = 13; NR, *N* = 29. **i** Plasma leptin level, CS, *N* = 5; FX, *N* = 7; IM, *N* = 7; NR *N* = 15. Results are shown as means ± s.e.m. *P < 0.05, **P < 0.01, ***P < 0.001

Obesity-prone subgroups had significantly different body weight and accumulated higher food intake at the end of the experiment. Obesity-prone stressed animals treated with vehicle (obesity prone CS) were significantly lighter than obesity-prone FX and obesity-prone NR animals (Fig. 4a, respectively, *P* < 0.05 and *P* < 0.01, and SI Appendix, Table S2), and their accumulated food intake was also significantly lower (Fig. 4b, both at *P* < 0.05, and SI Appendix, Table S2). Food efficiency (food intake/Δ body weight) of the obesity-prone FX subgroup was significantly lower than the obesity-prone CS and obesity-prone NR subgroups, while obesity-prone IM subgroup was significantly lower than the obesity-prone NR subgroup (Fig. 4c, *P* < 0.05, *P* < 0.001, and *P* < 0.05, respectively, and SI Appendix, Table S2).

### Chronic stressed obesity prone saline-treated animals had lower lean and fat mass

Obesity-prone stressed animals treated with saline (obesity-prone CS) had significantly lower lean mass and fat mass in comparison to the obesity-prone NR subgroup, and lower lean mass than obesity-prone FX animals (respectively, *P* < 0.05, *P* < 0.01, *P* < 0.001, Fig. 4d, e, and SI Appendix, Table S2). The obesity-prone IM subgroup also had lower lean mass than the obesity-prone NR and the obesity-prone FX subgroups (respectively, *P* < 0.05 and *P* < 0.001, Fig. 4d, and SI Appendix, Table S2). However, there were no significant differences between the obesity-resistant subgroups in body weight, accumulated food intake, and food efficiency (SI Appendix, Table S2).

### Chronic stressed obesity prone saline-treated animals had worse bone features, and obesity prone FX-treated animals had enhanced bone features and axial length

Stressed animals treated with saline (obesity-prone CS) had lower BMD and BMC in the DXA scan, in comparison to the obesity-prone NR and obesity-prone FX subgroups (respectively, BMD: *P* < 0.01, *P* < 0.01, and BMC: *P* < 0.001, *P* < 0.001, Fig. 4f–g, and SI Appendix, Table S2). Obesity-prone FX-treated animals had increased axial length in comparison to the other obesity-prone subgroups (*P* *<* 0.01 for all comparisons, Fig. 4h, and SI Appendix, Table S2). Within obesity-resistant groups, there was no significant difference in axial length, fat mass, BMD, or BMC (SI Appendix, Table S2).

### Chronic stressed obesity prone FX-treated animals had increased leptin plasma levels and were associated with body composition outcomes

**Fig. 5:**
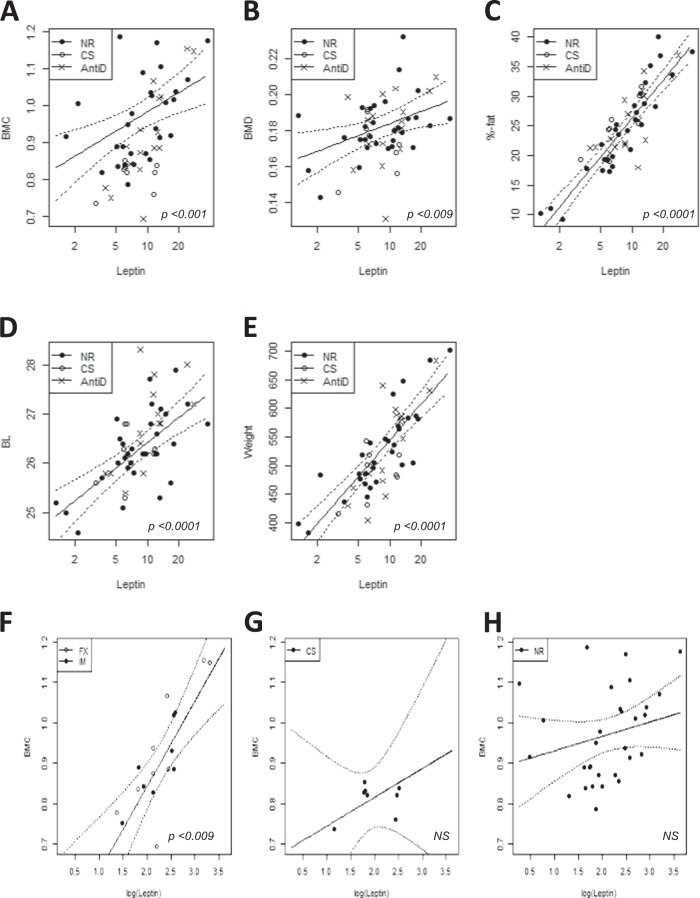
Associations between leptin and body composition outcomes **a** BMC, bone mineral content; **b** BMD, bone mineral density; **c** fat percentage; **d** BL, body length; **e** body weight. NR = non-restraint control group, *N* = 30; CS = chronic restraint stress (CRS) group treated with saline, *N* = 8; AntiD = CRS + antidepressant-treated group, *N* = 17. **f**–**h** The interaction between leptin and BMC with its treatment outcomes; **f** leptin levels were strongly associated with BMC in the fluoxetine- (FX) and imipramine- (IM) treated animals; leptin levels were not signifincantly associated in the **g** CS group and in the **h** NR group. AntiD = includes FX and IM

Within CRS obesity-prone subgroups, plasma leptin level was significantly elevated in the FX obesity-prone subgroup compared to the CS obesity-prone subgroup (*P* < 0.05, Fig. 4i, and SI Appendix, Table S2). Leptin was strongly associated with all five body composition outcomes: BMC, BMD, fat percentage, body length, and body weight (respectively, *P* = 0.001, *P* = 0.009, *P* < 0.0001, *P* < 0.0001, and *P* < 0.0001, Fig. 5a–e, and SI Appendix, Table S4). The interaction between leptin and antidepressant treatment was significant for BMC (*P* = 0.009, Fig. 5f–h, and SI Appendix, Table S4). We also found weak evidence for an interaction between triglycerides and antidepressant treatment in body length (*P* = 0.05, SI Appendix, Table S4).

### Triglyceride plasma levels were lower in chronic stressed saline- and FX-treated animals

In all the groups, we measured plasma lipid profile (SI Appendix, Fig. S2), and we found that only triglyceride level was significantly different; CS and FX groups had lower tryglyceride levels in comparison to the NR group (Fig. S3A, *P* < 0.001 and *P* < 0.05, respectively).

### Chronic stressed antidepressant-treated animals had increased lipolysis in white adipose tissue

The relative expression levels for the 8 target genes were quantified in epididymal fat pad samples using qPCR. No significant differences were observed between groups in the level of expression of any of the genes of interest except for the *Lipe* gene, for which the IM and FX groups were found to have a higher mean expression level than the NR group (Fig. S4, and SI Appendix, Table S5).

## Discussion

Weight gain is a common outcome of antidepressant treatment in the clinical setting, but several studies have shown that chronic administration of various antidepressants results in failure to gain weight or “paradoxical” weight loss in rats, especially at high doses^20,21^. Until recently, the lack of an appropriate animal paradigm caused gaps in the understanding of the pathways involved in antidepressant-induced weight gain in clinical populations. We have developed the SADIO model to help expand our understanding of the interface between obesity, MDD, and antidepressants. This model has allowed the understanding of the long-term impact of the combined effects of limited exposure to recurrent stress and antidepressant (2 weeks), followed by chronic intake of high-fat diet (>9 months) on body composition and bone morphology. In this study, we evaluated body weight recovery patterns, body composition, bone features, biochemical and gene expression measurements during the post-stress acclimation period, which is a recovery phase in which the animal is free from the stressor exposure^17^. In agreement with previous literature, 2 weeks of CRS induced significant weight loss in our animals (SI Appendix, Fig. S2)^22^. Within the studied timeframe, the control (CS) group did not attain full body weight recovery in comparison to the NR group, while both antidepressant-treated groups had much better body weight recovery. The ability of the SADIO model to recover from CRS-induced weight loss during the post-stress acclimation period could be referred to as a phenomenon of “catch-up growth”, which is growth that occurs after a period of growth-inhibiting conditions^23^. Our results suggest that CRS and antidepressant treatment resulted in a period of greater rate of catch-up growth during the post-stress acclimation period. The IM group made full body weight recovery and the FX group had an exacerbated body weight recovery, recovery weights of the FX groups were significantly larger than that of the CS group. These findings suggest that antidepressant-treated groups had better compensatory responses that are associated with induction of hyperphagia, elevated body weight, and repletion of energy reserves leading to super abnormal linear growth^23^.

We found that the obesity-prone FX animals had increased caloric intake, were lengthier and heavier, and had increased leptin levels, yet had significantly lower food intake ratio, suggesting that they had greater ability to store energy. The exacerbated weight gain in the FX group was associated with significant increase in lean mass, BMD, and BMC, and pronounced positive changes in bone cortical and trabecular morphology. Congruently, 9 months (endpoint) after stress and antidepressant use were ceased in the SADIO model, the obesity-prone FX subgroup had significantly greater body length in comparison to the obesity-prone NR and obesity-prone CS subgroups, implying that the propensity to gain weight can influence the rate of catch-up growth and bone allometry. On the other hand, although plasma leptin level was elevated in the obesity-prone FX subgroup in comparison to the obesity-prone CS subgroup, the obesity-prone FX subgroup did not have significant increase in fat mass; yet, the fat mass was significantly lower in the obesity-prone CS subgroup in comparison to the obesity-prone NR subgroup. Furthermore, the metabolic and lipid plasma profiles were not significantly elevated in the FX group, suggesting that there was no further metabolic/lipid dysregulation associated with FX-induced weight gain (SI Appendix, Fig. S3). Our gene expression data suggest that it is unlikely that adipose tissue inflammation and metabolism were meaningfully altered by antidepressant treatment in the SADIO paradigm, as we found no group differences in the expression of genes that indicate adipose tissue inflammation (*Tnf*) and insulin sensitivity (*Slc2a4, Adipoq* and *Pparg*). Moreover, the expression of a gene involved lipolysis (*Lipe*) was significantly elevated in the FX and IM groups, indicating that there may have been enhanced lipolysis activity in these groups.

The literature on the effect of long-term fluoxetine use on bone metabolism remains controversial. Ortuno et al.^24^ have shown that long-term FX treatment results in serotonin-dependent rise in sympathetic output that increases bone resorption sufficiently to counteract the local anti-resorptive effect. Our result shows that FX treatment and stress for 2 weeks in the context of long-term high-fat diet (over 276 days) resulted in increased body weight and length, accompanied by enhanced femoral bone features. Thus, the mechanism of SSRI action on bone after its discontinuation, in the presence of high-fat diet, is likely to be different from that of SSRI treatment alone, as our findings of elevated BV/TV and trabecular number are compatible with both reduced bone resorption, intensified bone formation (greater trabecular thickness) and increased cortical bone features and bone/body length suggesting enhanced bone growth. Regardless of treatment duration, antidepressant treatment most likely played a role during catch-up growth in the post-stress acclimation period. Furthermore, there was a strong correlation between plasma leptin level and BMC in the antidepressant-treated groups, which was absent in the NR or the CS control groups. Both, peripheral and central effects of leptin have been described to play a role in bone formation^25,26^. Peripheral leptin has been reported to have direct bone anabolic function and increase osteoblast number and activity^27^; however, via an indirect hypothalamic-mediated pathway, centrally leptin inhibits bone formation by regulating both phases of bone remodeling, resorption and, formation^28–30^, which suggest that short-term antidepressant treatment either lessened the central inhibitory and/or enhanced the peripheral role of leptin in bone formation in the SADIO model, resulting in enhanced bone features.

Weight gain can be induced by mechanisms other than increased fat mass, and growth induced via the GH/IGF1; somatotropic axis is an important factor in weight gain. Wu et al. (2009) demonstrated that the over-expression of systemic IGF1 resulted in enhanced morphological bone features that resemble those of the SADIO model, such as increased cortical thickness and total bone mineral density, and enhanced trabecular bone volume^31,32^. However, we found no significant differences in the endpoint IGF-1 plasma level between the groups; nevertheless, the secretion of GH declines with age and GH/IGF-1 levels may have been different in the earlier stage of weight gain^33,34^.

In this study, we show that IGF1 mRNA levels in the liver are elevated in the CS group than in comparison to the NR group. Although there was no significant difference in systemic IGF-1 level between the groups, significantly lower IGF1 mRNA level in liver in NR group could provide explanation that NR group did not have enhanced bone morphological features compared to CS group. Sjögren et al. (2002) showed that inactivation of liver IGF1 induced a decrease in cortical cross-sectional area and periosteal circumference in the mid-diaphyseal region of the femur and was associated with weaker bone in the mechanical loading test^35^. Thus, weight gain of NR group could be due to diet-induced obesity rather than the growth associated with enhanced bone morphological features.

Previous findings have suggested that short-term FX treatment induces weight loss^36^. While on the one hand, serotonin receptors 5-HT_2A_ and 5-HT_2c_ have been proposed to play a significant role in the inhibition of appetite and food intake^37^, on the other hand, long-term FX treatment has been shown to lead to weight gain^10–12,36,38^. Despite numerous studies, the effects of FX treatment in weight regulation remain elusive. Adherence to antidepressant treatment is low; in a large European study of 7525 patients, 56% abandoned treatment within 4 months, and weight gain was a major side-effect of antidepressant. Antidepressants including mirtazapine^39^, paroxetine^40^, and amitriptyline^41^ have been associated with long-term weight gain, and meta-analysis showed that amitriptyline and paroxetine induced the greatest weight gain in periods over 4 months;^36^ thus, future investigations could address the effects of these antidepressant drugs on weight regulation in the SADIO model.

There are several limitations and future directions for this study. Although our study indicated that stress and antidepressant (SAD)-induced weight gain is associated with growth, we could not clearly show whether the somatotropic axis is involved. We only measured GH/IGF-1 levels at the end of the experiment and as animals got older, the growth rate was sustained at much slower rate than in earlier periods. In the future, pulsatile GH levels in plasma should be characterised during an earlier period when a higher growth rate is observed, in order to obtain a better understanding of the mechanism behind SAD-related weight gain.

In the SADIO model, 6 h of CRS unavoidably may have resulted in food deprivation. Although restraint stress was applied during the light cycle, the effects of food restriction experience on diet-induced weight gain and bone formation cannot be ruled out. For future studies, one additional group with 6 h of food deprivation should be included in the experimental design.

Our findings of decreased bone structural features after stress are compatible with those described for major depression and chronic stress-induced depressive-like behavior leading to bone loss^42–45^. Furthermore, Yirmiya R et al. (2006) have shown that chronic stress decreased osteoblast numbers and bone formation via activation of the sympathetic nervous system, and co-treatment with IM attenuated bone loss only in animals that improved from depressive-like behaviors (responders)^45^. However, to our knowledge this is the first report on the long-lasting effects of short-antidepressant treatment in body growth and bone features, which may manifest dissimilarly in subsets of the population with different propensity to gain weight while consuming high-fat diets. As osteoporosis is a condition with significant morbidity and increasing prevalence due to the aging population^46,47^, our findings demonstrating that environmental factors may modulate the role of leptin, may contribute to the understanding of aspects relevant to the heterogeneity and treatement approaches of this condition^48^.

This study has several key findings: (1) Weight gain after recurrent stress and short-term antidepressant treatment followed by diet-induced obesity is different from that of the classic diet-induced obesity. (2) Short-term antidepressant treatment has long-term consequences, and ameliorated the effects of chronic stress on body growth (FX), had long lasting effects in anxiety-like behavior (FX) and adipose tissue gene expression (FX and IM), and affected bone allometric processes that lead to exacerbated bone growth (FX/axial and IM/femur) and enhanced bone structural features (FX). (3) Those effects on body growth and bone features are highly associated with leptin levels. (4) There is a significant interaction between short-term antidepressant treatment and leptin, that likely lessened the central role of leptin in inhibiting bone formation; thus, pharmacological agents may influence the role of leptin in long-term bone regulation. Clinical studies monitoring body weight, caloric intake, and body and bone features after antidepressant discontinuation are needed to confirm our observations in the clinical setting.

## Supplementary information

Supplementary Information
