# Supplementary material for: Short-term antidepressant treatment has long-lasting effects, and reverses stress-induced decreases in bone features in rats
Source: Transl Psychiatry. 2019 Jan 16;9:10. doi: 10.1038/s41398-018-0351-z (PMC6341077; doi:10.1038/s41398-018-0351-z)
Supplement: Supplementary file 1 — Supplementary Information [file 41398_2018_351_MOESM1_ESM.docx]

**Supplementary material Methods**

**
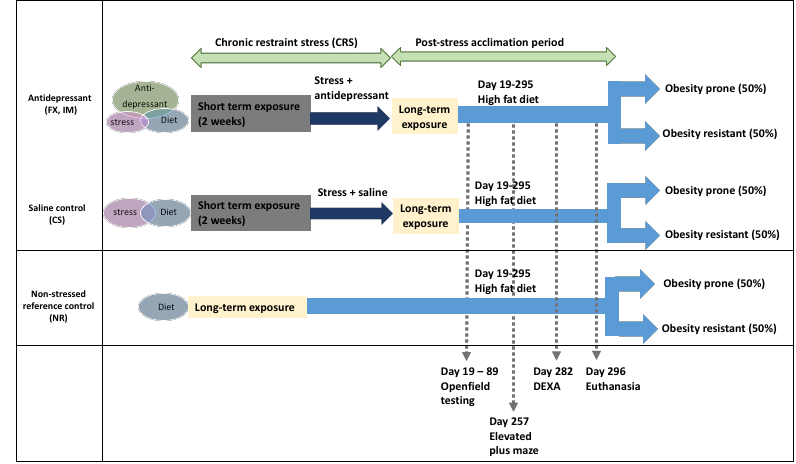
**

**Fig. S1. Overview of the experimental protocol of SADIO paradigm.**

**Dual energy x-ray absorptiometry (DXA) for body composition analysis and body length measurements**

Subsets of obesity prone (OP) and obesity resistant (OR) animals were submitted to DXA on experimental day 280 two weeks prior to euthanasia on experimental day 296. The body composition profile was determined using the Lunar imaging software (ver.1.46, GE Lunar, WI, USA) and regions of interest (ROI) were chosen. ROI included abdominal region from the last rib to pelvic bone (Figure 3-4). Parameters of bone mineral density (BMD), bone mineral content (BMC), lean tissue, and percentage of fat tissue were measured. We scanned each ROI five times and the average measurement per individual animal was calculated. The coefficient of variation within an individual animal was less than 5%.

**Tissue and plasma collection**

Rats were euthanized between 10:00–12:00 h on experimental day 296. Upon decapitation, truncal blood was collected in tubes of 0.5 M EDTA. Tubes were mixed, centrifuged at 3000 *g* for 17 minutes at 4 °C, and plasma were stored at –70 °C. Epididymal fat pads and liver tissue were dissected, frozen immediately, and stored at –70 °C. Hind femurs were collected in 4% paraformaldehyde, stored for 4 days, and then transferred to 70% ethanol at 4 °C.

**Microtomography (Micro-CT)**

in order to extract the region of interest and discriminate bone and non-bone areas. We chose a transverse plane slice where growth plate was just about to merge as a “landmark slice”, and regions of interest were chosen relative to this slice. For trabecular bone analysis, a 2 mm section of interest (94 slices) was chosen in offset of 2 mm (94 slices) away from the landmark slice. For cortical bone analysis, a 3.01 mm section of interest (141 slices) was chosen in offset of 10.01 mm (469 slices) away from the landmark slice.

**qPCR**

**Primer pairs:**

| ***Gene*** | ***Forward*** | ***Reverse*** |
| --- | --- | --- |
| ***Igf1*** | 5’CACATCTCTTCTACCTGGCACTC3’ | 5’GGATGGAACGAGCTGACTTTGTA3’ |
| ***Gapdh*** | 5’CCATTCTTCCACCTTTGATGCT3’ | 5’TGTCATACCAGGAAATGAGCTTCA3’ |
| ***Tnf*** | 5’AAGGAGGAGAAGTTCCCAAATG3’ | 5’GCTTGGTGGTTTGCTACGA3’ |
| ***Slc2a4*** | 5’AGTATGTTGCGGATGCTATGG3’ | 5’CTCTGGTTTCAGGCACTCTTAG3’ |
| ***Adipoq*** | 5’GGAAACTTGTGCAGGTTGGA3 | 5’CTTAGGACCAAGAACACCTGC3’ |
| ***Pparg*** | 5’GGATGTCTCACAATGCCATCA3’ | 5’GTTCAGCTGGTCGATATCACTG3’ |
| ***Lipe*** | 5’CCTCCAAACAGAAACCCGATT3’ | 5’GAATGCCGAGGCTGTATCC3’ |
| ***Lpl*** | 5’CTGATTCCTGGATTAGCAGACTC3’ | 5’CATTCCTGTCACCGTCCATC3’ |
| ***Fasn*** | 5’CCTTGGGAGTCAAAGTGTCAG3’ | 5’CGATGAGGGCAATCTGGATG3’ |
| ***Ppargc1a*** | 5’AAGAGCGCCGTGTGATTTA3’ | 5’AACCATAGCTGTCTCCATCATC3’ |
| ***Actb*** | 5’CTGACTGACTACCTCATGAAGATCCT3’ | 5’CTTAATGTCACGCACGATTTCC3’ |
| ***Rps18*** | 5’TTCAGCACATCCTGCGAGTA3’ | 5’TTGGTGAGGTCAATGTCTGC3’ |

**RNA extraction and cDNA synthesis**

Total RNA was isolated from the liver and epididymal fat pad tissues with RNeasy Lipid Mini Kit (Qiagen, Chadstone Centre, VIC, Australia) or PureLink RNA MiniKit (ThermoFisher Scientific, Waltham, MA, USA) using DNase digestion. Reverse transcription was performed using OMNISCRIPT RT KIT (Qiagen) or iScript cDNA Synthesis Kit (Bio-Rad Laboratories Pty, Ltd, Gladesville, NSW, Australia) with Oligo (dT) primers (Invitrogen, Mount Waverley, VIC, Australia). Rat *Gapdh,* glyceraldehyde-3-phosphate dehydrogenase, or the geometric mean of actin beta (*Actb)* and ribosomal protein S8 (*Rps8*) Ct (threshold cycle) values were chosen as endogenous reference controls. Reactions were performed using Power SYBR Green PCR Master Mix (Applied Biosystems), the 7900HT Fast Real Time PCR system (Applied Biosystems, Foster city, USA) or the QuantStudio 7 Flex Real-Time PCR System (Applied Biosystems). The standard curve method (liver) or the relative quantification method (adipose) was used to calculate relative gene expression levels.

**ELISA protocols**

The following kits were used in this experiment. 1) Rat IGF1 Immunoassay (Quantikine, R&D systems, Braeside, VIC, Australia), 2) Rat Leptin ELISA (Merck Millipore, Billerica), 3) Triglyceride Quantitation kit (Abcam, Cambridge, UK), 4) Cholesterol/Cholesteryl Ester Quantitation kit (Abcam, Cambridge, UK), 5) Rat vanillylmandelic acid (Cusabio, China), and 5) Rat/mouse growth hormone ELISA, (Merck Millipore, Billerica).

**Quantification of GH concentration in pituitary samples**

*Total protein extraction* Pituitary samples were homogenized in 200 µL of tissue extraction reagent I (Invitrogen, Camarillo, USA) with protease inhibitor cocktail (Sigma, St Louis, USA) added. Samples were centrifuged at 10,000 RPM (9,400 *g*) for 5 minutes, and tissue debris were pelleted. Supernatants were collected and diluted 1:128 for the quantification of total protein (Bio-Rad protein assay, Bio-Rad, Hercules, USA). Assay was performed following the standard procedure for microtiter plates. 10 µL of each sample and standards were mixed with 200 µL of diluted dye reagent and incubated at room temperature for 5 minutes. A standard curve was generated in the concentration range between 0.0313 – 0.5 mg/mL and samples were tested in duplicates. Absorbance was measured at the wavelength of 595 nm. Total protein extracts from each pituitary were diluted to concentration of 4mg/mL. Samples were further diluted in the dilution range of 1:8000 – 1:24000 by two step serial dilution. GH concentration was determined using immunoassay kit following the manufacturer’s protocol (rat/mouse growth hormone ELISA, Merck Millipore, Billerica).

**Lipid/metabolite quantification**

***Triglyceride (TG) quantification***

TG in plasma samples was determined by a colorimetric assay using the Triglyceride Quantitation kit (Abcam, Cambridge, UK). TG was converted to free fatty acids and glycerol in the presence of lipase. Glycerol was oxidized and reacted with the probe resulting in a color change detected at the wavelength of 570 nm. Samples were tested in duplicates and diluted in the range of 1:6 to 1:16. TG concentration was calculated by amount of TG (µg)/volume of sample (µL) per well.

***Cholesterol quantification***

Cholesterol concentration in plasma samples was determined by a colorimetric assay using the Cholesterol/Cholesteryl Ester Quantitation kit (Abcam, Cambridge, UK). Cholesterol esters in plasma were hydrolyzed to cholesterol in the presence of cholesterol esterase. Cholesterol was oxidized into H_2_O_2_ and reacted with the cholesterol probe resulting in a color change, detected at the wavelength of 570 nm. Samples were diluted in 1:30 and tested in duplicates. Cholesterol concentration was calculated by amount of cholesterol (µg)/volume of sample (µL) per well.

***FFA quantification***

FFA in plasma samples was determined by a colorimetric assay by using the free fatty acid Quantitation kit (Abcam, Cambridge, UK). FFA was converted to CoA derivatives in the presence of Acyl-CoA synthetaze, resulting in a color change and detected at the wavelength of 570 nm. Samples were tested in duplicated and diluted in the range of 1:4. Free fatty acid concentration was calculated by amount of free fatty acid (µg)/volume of sample (µL) per well.

***VMA immunoassay***

VMA levels in plasma samples were determined by immunoassay following the manufacturer’s protocol (Rat vanillylmandelic acid, Cusabio, China). A standard curve range was generated in the concentration range between 0–500 ng/mL. Samples were diluted in 1:200 and tested in duplicates, and the coefficient of variation between duplicates was below < 15%.

**Statistical analysis**

In each period the weights models were:

$$y_{ij}= \beta_{1}+ \left( \beta_{0}-\beta_{1} \right)\exp\left( -\beta_{2}\left( x_{ij}-a_{k} \right) \right)+\epsilon_{ij}$$

where *i* index the rats,$x$ are rat ages (days), the $\beta_{0}$ is the weight at the first day $a_{k}$ of each period, $\beta_{1}$ is the asymptotic weight, and $\beta_{2}$ is the decay rate. All three growth parameters varied by treatment (NR v FX v IM v CS), obesity prone weight groups (OP v OR), the pairwise weight group-treatment interaction (fixed effects), and random effects per rat. The overall comparison across treatment groups was the sub-model with the weight group fixed effects excluded. Log-likelihood ratio test of nested sub-models was used to assess the interaction between weight-groups and treatment. Weights were assumed to be autocorrelated AR. We chose to separate the periods at day 60 as rat weight change appeared to have finished transitioning from a high recovery growth rate period (days 20-60) to a more sedate growth rate (post day 60). Post-CRS weight gain was estimated from day 20 as

$$\Delta y_{ij}=\left( \hat{\beta}_{1}^{2}-\hat{\beta}_{0}^{2} \right)\left( 1- exp\left( -\hat{\beta}_{2}^{2}\left( x_{ij}-60 \right) \right) \right)+ \left( \hat{\beta}_{0}^{2}-\hat{\beta}_{0}^{1} \right),$$

where the superscript refers to the first (days 20-60) and second (days > 60) periods. Confidence intervals of the difference between treatment groups in weight gain were estimated at day 300 using a 999 replicate basic boostrap with rat as the resampling unit.

Associations between the five body composition outcomes (BMC, BMD, % fat, body length, body weight) with four biochemical measures (log-transformed plasma leptin, total cholesterol, triglyceride, and fatty acids levels) were tested using linear regressions. For each body composition outcome the interaction between biochemical measures and treatment group (FX and IM vs RC vs NR) were analyzed; F tests of nested linear models were employed.

**Fig. S2. Body weight and accumulated food intake immediately after the CRS period**. The chronic stress groups treated with saline (CS, n=12), fluoxetine (FX, n=14) and imipramine (IM, n=13), and non-stressed reference (NR, n=30) group. Results are shown as means ± SEM. ***, *P*<0.001; *, *P*<0.05.

**Fig. S3. Comparison of plasma lipid profile between groups**. (A) Triglyceride (B) Cholesterol (C) Free Fatty acid, and (D) Vanillylmandelic acid (VMA). Treatment groups were Control Saline (CS, n=10), Fluoxetine (FX, n=14), Imipramine, (IM, n=13), and non-restraint (NR, n=26-30). Results are shown as means ± s.e.m. *, *P* <0.05; ***, *P*<0.001.


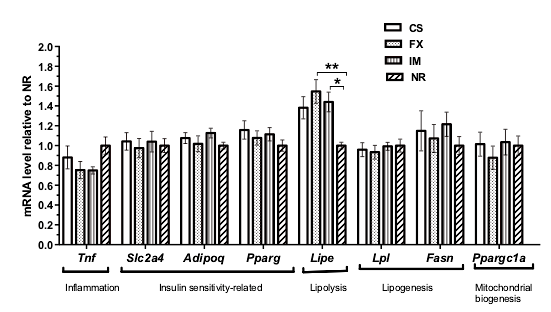


**Fig. S4.** Relative gene expression levels of 8 genes of interest in epididymal fat pad samples using quantitative PCR. The expression of genes reflective of adipose tissue inflammation (*Tnf*, tumour necrosis factor), insulin sensitivity-related (*Slc2a4*, glucose transporter type 4; *Adipoq*, adiponectin; *Pparg*, peroxisome proliferator activated receptor γ), lipolysis (*Lipe*, lipase E, hormone sensitive type), lipogenesis (*Lpl*, lipoprotein lipase; *Fasn*, fatty acid synthase), and mitochondrial biogenesis (*Ppargc1a*, peroxisome proliferator activated receptor gamma co-activator 1α) are shown. A significant difference was found in (*Lipe*) expression between the NR, non-restraint, N=11, group and the chronic restraint stress (CRS) imipramine-treated, IM, N=11, and the CRS fluoxetine-treated, FX, N=12, groups. CS, CRS saline treated (N=8). No other significant differences were observed. mRNA, messenger RNA. Results are shown as means ± s.e.m. **P* ≤ 0.05, ***P* ≤ 0.01.

**Table S1:** **Non-linear mixed effects regression analysis on difference of body weight between treatment groups.**

| **(A)** |  |  | **Period I (day 20-60)** | |  | **Period II (day >60)** | |
| --- | --- | --- | --- | --- | --- | --- | --- |
|  |  |  | **Est [95%CI]** | **p-value** | | **Est [95%CI]** | **p-value** |
| $\hat{\beta}_{1}$ |  | NR | 446.7 [432.2, 461.2] | <0.0001 | | 559.4 [530.7, 588.1] | <0.0001 |
|  |  | CSvNR | -56.5 [-82.8, -30.2] | <0.0001 | | -22.3 [-76.0, 31.4] | 0.41 |
|  |  | FXvNR | -33.3 [-58.4, -8.2] | 0.008 | | 52.9 [ 1.9, 103.8] | 0.04 |
|  |  | IMvNR | -39.3 [-64.9, -13.6] | 0.002 | | -5.6 [-57.1, 46.0] | 0.83 |
| $\hat{\beta}_{0}$ |  | NR | 314.3 [307.7, 321.0] | <0.0001 | | 431.8 [419.6, 444.0] | <0.0001 |
|  |  | CSvNR | -36.8 [-49.2, -24.4] | <0.0001 | | -48.8 [-71.4, -26.2] | <0.0001 |
|  |  | FXvNR | -39.4 [-51.1, -27.7] | <0.0001 | | -31.6 [-53.0, -10.1] | 0.003 |
|  |  | IMvNR | -37.9 [-49.9, -25.9] | <0.0001 | | -32.6 [-54.6, -10.6] | 0.003 |
| $\log\hat{\beta}_{2}$ |  | NR | -3.05 [-3.17, -2.94] | <0.0001 | | -5.06 [-5.12, -5.01] | <0.0001 |
|  |  | CSvNR | 0.25 [ 0.04, 0.46] | 0.02 | | -0.16 [-0.29, -0.03] | 0.01 |
|  |  | FXvNR | 0.18 [-0.02, 0.38] | 0.07 | | -0.31 [-0.41, -0.21] | <0.0001 |
|  |  | IMvNR | 0.20 [ 0.00, 0.41] | 0.05 | | -0.01 [-0.10, 0.09] | 0.87 |

| **(B)** |  | **Weight diff. [95%CI]** | **p-value** |
| --- | --- | --- | --- |
| **All** | CS-NR | 0.2 [-30.2, 31.3] | 1.00 |
|  | FX-NR | 49.6 [5.4, 92.2] | 0.02 |
|  | IM-NR | 26.6 [-3.9, 52.9] | 0.12 |
|  | FX-CS | 49.4 [6.7, 91.7] | 0.03 |
|  | IM-CS | 26.4 [-3.2, 55.6] | 0.10 |
|  | IM-FX | -23.0 [-64.6, 19.4] | 0.32 |
| **OP** | CS-NR | -19.9 [-60.0, 12.2] | 0.20 |
|  | FX-NR | 69.3 [25.2, 102.8] | 0.004 |
|  | IM-NR | 16.0 [-17.6, 48.8] | 0.43 |
|  | FX-CS | 89.2 [47.5, 130.3] | <0.0001 |
|  | IM-CS | 35.9 [1.4, 72.0] | 0.04 |
|  | IM-FX | -53.3 [-87.4, -14.5] | 0.01 |
| **OR** | CS-NR | 9.8 [-21.6, 42.5] | 0.59 |
|  | FX-NR | 36.3 [-1.9, 71.5] | 0.06 |
|  | IM-NR | 35.1 [14.8, 51.9] | <0.0001 |
|  | FX-CS | 26.5 [-18.1, 68.9] | 0.24 |
|  | IM-CS | 25.4 [-5.8, 51.1] | 0.14 |
|  | IM-FX | -1.2 [-36.6, 35.4] | 0.86 |

| **(C)** |  |  | **Period I (day 20-60)** | | **Period II (day >60)** | | |
| --- | --- | --- | --- | --- | --- | --- | --- |
|  |  |  | **Est [95%CI]** | **p-value** | **Est [95%CI]** | **p-value** | |
| $\hat{\beta}_{1}$ | OP | NR | 421.5 [407.1, 435.8] | <0.0001 | 500.1 [ 469.0, 531.1] | <0.0001 | |
|  |  | CSvNR | -30.8 [-59.0, -2.6] | 0.03 | 22.1 [ -44.7, 88.9] | 0.51 | |
|  |  | FXvNR | -47.7 [-72.0, -23.3] | <0.0001 | 129.1 [ 54.5, 203.8] | 0.0005 | |
|  |  | IMvNR | -28.7 [-54.4, -3.0] | 0.03 | 1.7 [ -54.4, 57.7] | 0.95 | |
|  | OPvOR | NR | 49.1 [ 28.6, 69.6] | <0.0001 | 112.1 [ 69.0, 155.1] | <0.0001 | |
|  |  | CSvNR | -50.6 [-89.9, -11.4] | 0.01 | -74.5 [-164.9, 15.8] | 0.10 | |
|  |  | FXvNR | 29.6 [ -5.6, 64.8] | 0.09 | -74.1 [-165.7, 17.5] | 0.11 | |
|  |  | IMvNR | -22.5 [-58.3, 13.2] | 0.21 | -13.9 [ -91.4, 63.5] | 0.72 | |
| $\hat{\beta}_{0}$ | OP | NR | 308.2 [298.4, 318.0] | <0.0001 | 411.3 [ 398.0, 424.5] | <0.0001 | |
|  |  | CSvNR | -28.0 [-47.1, -8.9] | 0.003 | -36.6 [ -62.4, -10.8] | 0.005 | |
|  |  | FXvNR | -43.3 [-60.3, -26.2] | <0.0001 | -43.4 [ -66.3, -20.5] | 0.0002 | |
|  |  | IMvNR | -31.9 [-49.8, -13.9] | 0.0004 | -24.5 [ -48.7, -0.3] | 0.04 | |
|  | OPvOR | NR | 13.7 [ 0.1, 27.3] | 0.04 | 39.3 [ 20.9, 57.8] | <0.0001 | |
|  |  | CSvNR | -19.9 [-46.9, 7.1] | 0.14 | -24.4 [ -60.7, 11.9] | 0.18 | |
|  |  | FXvNR | 9.6 [-14.3, 33.5] | 0.42 | 25.8 [ -6.4, 58.0] | 0.11 | |
|  |  | IMvNR | -12.6 [-37.2, 11.9] | 0.30 | -17.7 [ -50.9, 15.5] | 0.29 | |
| $\log\hat{\beta}_{2}$ | OP | NR | -3.00 [-3.10, -2.90] | <0.0001 | -5.00 [-5.10, -4.80] | <0.0001 | |
|  |  | CSvNR | -0.10 [-0.30, 0.20] | 0.61 | -0.60 [-0.90, -0.20] | 0.0005 | |
|  |  | FXvNR | 0.20 [ 0.10, 0.40] | 0.004 | -1.10 [-1.40, -0.80] | <0.0001 | |
|  |  | IMvNR | 0.20 [ 0.00, 0.40] | 0.02 | 0.20 [ 0.00, 0.40] | | 0.02 |
|  | OPvOR | NR | -0.20 [-0.40, -0.10] | 0.003 | -0.10 [-0.30, 0.00] | | 0.09 |
|  |  | CSvNR | 0.60 [ 0.30, 0.90] | <0.0001 | 0.30 [ 0.00, 0.70] | | 0.06 |
|  |  | FXvNR | -0.10 [-0.30, 0.10] | 0.26 | 1.10 [ 0.80, 1.40] | | <0.0001 |
|  |  | IMvNR | 0.10 [-0.10, 0.30] | 0.49 | -0.30 [-0.50, -0.10] | | 0.001 |

(A) Non-linear mixed effects regression estimates for periods day 20-60 and >60, for the model with only treatment fixed effects. (B) Non-linear mixed effects regression estimates for periods day 20-60 and >60, for the model with treatment, obesity prone groups and their pairwise interaction as fixed effects. (C) Estimated differences in weight change between days 20 and 300 with bootstrapped 95% confidence intervals for all group, OP group and OR group. CS, control saline group, FX, fluoxetine-treated group; IM, imipramine-treated group, NR, non-restraint reference group; OP, obesity prone; OR, obesity resistant.

| Table S2. Body, bone allometric, and biochemical measurements, and analysis of anxiety and locomotor activity for the chronic restraint stress groups during the post-restraint stress period. | | | | | |
| --- | --- | --- | --- | --- | --- |
|  | **OP CS** | **OP FX** | **OP IM** | **OP NR** | **Significance** |
| Body weight (g) | 506.10 ± 12.07 | 607.30 ± 14.86 | 544.10 ± 10.58 | 580.50 ± 14.49 | *P*=0.003 |
| Accumulated food intake (g) | 4759.00 ±  136.80 | 5335.00 ±  96.26 | 4920.00 ±  81.48 | 5277.00 ±  110.20 | *P*=0.01 |
| Food intake ratio | 20.37 ± 0.89 | 16.22 ± 0.5 | 17.90 ± 0.63 | 21.65 ± 0.84 | *P*=0.001 |
| Lean mass (g) | 116.90 ±  2.97 | 144.50 ±  4.82 | 118.70 ±  3.23 | 134.10 ±  2.49 | *P*=0.001 |
| Fat mass (g) | 34.69 ±  3.52 | 46.58 ±  6.62 | 41.56 ±  4.10 | 58.01 ±  2.90 | *P*=0.005 |
| BMC (g) | 1.81 ±  0.069 | 2.32 ±  0.11 | 2.07 ±  0.063 | 2.25 ±  0.040 | *P*=0.001 |
| BMD (g/cm^2^) | 0.1385 ±  0.0033 | 0.1713 ±  0.0036 | 0.1582 ±  0.0024 | 0.17 ±  0.0046 | *P*=0.001 |
| Body length (cm) | 26.53 ±  0.16 | 27.74 ±  0.20 | 26.85 ±  0.050 | 26.82 ±  0.16 | *P*=0.002 |
| Leptin (ng/mL) | 7.93 ± 1.31 | 15.82 ± 2.81 | 11.77 ± 0.80 | 14.20 ± 2.08 | *P*<0.05 (CRS) |
|  | **OR CS** | **OR FX** | **OR IM** | **OR NR** | **Significance** |
| Body weight (g) | 450.90 ± 14.68 | 473.00 ± 20.30 | 487.50 ±  7.43 | 466.00 ±  9.34 | *NS* |
| Accumulated food intake (g) | 4446.00 ±  189.30 | 4645.00 ±  162.10 | 4820.00 ±  76.83 | 4678.00 ±  74.73 | *NS* |
| Food intake ratio | 10.34 ±  0.50 | 9.78 ±  0.18 | 9.85 ±  0.16 | 10.33 ±  0.30 | *NS* |
| Lean mass (g) | 112.60 ±  2.11 | 118.50 ±  4.71 | 119.00 ±  3.00 | 123.40 ±  3.16 | *NS* |
| Fat mass (g) | 30.62 ±  2.89 | 30.57 ±  1.03 | 33.56 ±  1.93 | 25.77 ±  2.19 | *NS* |
| BMC (g) | 1.84 ±  0.052 | 2.01 ±  0.15 | 1.89 ±  0.029 | 2.02 ±  0.084 | *NS* |
| BMD (g/cm^2^) | 0.13 ±  0.005 | 0.15 ±  0.009 | 0.14 ±  0.002 | 0.15 ±  0.004 | *NS* |
| Body length (cm) | 25.85 ±  0.24 | 25.85 ±  0.21 | 26.15 ±  0.17 | 25.58 ±  0.18 | *NS* |
| Leptin (ng/mL) | 7.70 ± 1.70 | 8.14 ± 0.99 | 8.85 ± 1.78 | 6.28 ± 1.05 | *NS* |
|  | **CS** | **FX** | **IM** | **NR** | **Significance** |
| Femur length (mm) | 42.43 ±  0.17 | 43.17 ±  0.41 | 43.62 ±  0.27 | 43.16 ±  0.27 | *P*=0.04 |
| Ratio *Igf1*/*Gapdh* mRNA | 2.048 ±  0.34 | 2.12 ±  0.35 | 2.34 ±  0.15 | 1.02 ±  0.26 | *P*=0.001 |
| Plasma IGF1 (ng/mL) | 859.40 ±  43.36 | 996.10 ±  37.65 | 924.10 ±  50.99 | 938.00 ±  35.57 | *NS* |
| Pituitary GH (mg/mL) | 198.20 ± 29.69 | 271.10 ± 40.65 | 244.60 ± 55.74 | 196.80 ± 23.22 | *NS* |
| Leptin (ng/mL) | 7.83 ± 1.28 | 15.41 ± 2.69 | 11.51 ± 0.80 | 13.76 ± 1.99 | *P*<0.05 |
| OFT CD/TD ratio | 0.091 ±  0.0054 | 0.11 ±  0.0055 | 0.10 ±  0.0065 |  | *P* <0.05 |
| EPM open/closed arm ratio | 0.030 ±  0.0067 | 0.076 ±  0.015 | 0.031 ±  0.0073 |  | *P* = 0.007 |
| EPM Number of entries to open arm | 2.09 ±  0.37 | 4.08 ±  0.50 | 2.64 ±  0.54 |  | *NS* |

Values are presented as mean ± SEM (standard error of the mean). Group comparison was performed using one-way ANOVA or the non-parametric Kruskal-Wallis test when appropriate. OP, obesity prone; OR, obesity resistant; CS, control saline group; FX, fluoxetine treated group; IM, imipramine treated group, NR, non-restraint reference group; g, grams; BMC, bone mineral content; cm, centimeters; BMD, bone mineral content, mm, millimeters; *Igf1*/IGF1, insulin-like growth factor 1; mRNA, messenger ribonucleic acid; GAPDH, glyceraldehyde 3-phosphate dehydrogenase; GH, growth hormone; ng, nanograms; mg, milligrams; mL, milliliters, OFT, open field test; CD/TD, center to total distance ratio; EPM, elevated plus maze.

| Table S3. Micro-CT analysis. Trabecular bone analysis parameters | | | | |  |
| --- | --- | --- | --- | --- | --- |
|  | **CS** | **FX** | **IM** | **NR** | **Significance** |
| BV/TV (%) | 17.44 ± 1.521 | 22.01 ± 1.450 | 20.56 ± 1.142 | 16.23 ± 0.950 | *P*=0.003 |
| Trabecular thickness (mm) | 0.201 ± 0.008 | 0.216 ± 0.007 | 0.209 ± 0.006 | 0.187 ± 0.006 | *P*=0.01 |
| Trabecular separation (mm) | 0.771 ± 0.022 | 0.820 ± 0.027 | 0.834 ± 0.018 | 0.809 ± 0.016 | *NS* |
| Trabecular number (1/mm) | 0.858 ± 0.055 | 1.011 ± 0.043 | 0.9786 ± 0.036 | 0.856 ± 0.034 | *P*=0.02 |
| T.Pm (mm) | 14.51 ± 0.188 | 14.88 ± 0.207 | 14.72 ± 0.264 | 14.44 ± 0.136 | *NS* |
| B.Pm (mm) | 23.63 ± 0.39 | 24.05 ± 0.437 | 24.01 ± 0.548 | 23.48 ± 0.262 | *NS* |
| B.Pm-T.Pm (mm) | 9.117 ± 0.208 | 9.174 ± 0.240 | 9.288 ± 0.292 | 9.044 ± 0.131 | *NS* |
| Ct.Ar ($\mathbf{mm}^{\mathbf{2}}$) | 9.207 ± 0.1833 | 9.963 ± 0.236 | 9.458 ± 0.223 | 9.104 ± 0.135 | *P*=0.009 |
| C.Th (mm) | 0.779 ± 0.01 | 0.829 ± 0.013 | 0.789 ± 0.012 | 0.775 ± 0.007 | *P*=0.002 |
| MMI ($\mathbf{mm}^{\mathbf{4}}$) | 29.59 ± 1.404 | 33.23 ± 1.733 | 31.48 ± 1.923 | 28.85 ± 1.024 | *NS* |

Values are presented as mean ± SEM (standard error of the mean). CS, control saline group, FX, fluoxetine treated group; IM, imipramine treated group, NR, non-restraint reference group; (BV/TV) %, percentage of trabecular bone volume/total volume; Trabecular thickness; (mm), millimeters; Trabecular separation; Trabecular number. Cortical bone analysis parameters: B.Pm, Periosteal perimeter; T.Pm, endosteal perimeter; B.Pm-T.Pm, difference between periosteal and endosteal perimeters; Ct.Ar, mean cross-sectional bone area; C.Th, cross-sectional bone thickness; MMI, mean moment polar inertia of cortical bone. Results are shown as means ± SD. Group comparison was performed using one-way ANOVA or the non-parametric Kruskal-Wallis test when appropriate.

**Table S4. Leptin association with body composition outcomes.**

| **Outcome** | **Predictor** | **Direct** | **Interaction** |
| --- | --- | --- | --- |
| BMC | Leptin (ng/mL) | 0.001 | 0.009 |
| BMC | Cholesterol (ug/uL) | 0.65 | 0.44 |
| BMC | Triglyceride (nmol/uL) | 0.05 | 0.78 |
| BMC | Igf1 mRNA | 0.36 | 0.68 |
| BMC | Fatty acid (nmol/ul) | 0.54 | 0.25 |
| BMC | Vanillylmandelic acid (ng/mL) | 0.18 | 0.43 |
| BMD | Leptin (ng/mL) | 0.009 | 0.74 |
| BMD | Cholesterol (ug/uL) | 0.56 | 0.58 |
| BMD | Triglyceride (nmol/uL) | 0.1 | 0.7 |
| BMD | Igf1 mRNA | 0.41 | 0.25 |
| BMD | Fatty acid (nmol/ul) | 0.92 | 0.28 |
| BMD | Vanillylmandelic acid (ng/mL) | 0.91 | 0.51 |
| Fat % | Leptin (ng/mL) | <0.0001 | 0.49 |
| Fat % | Cholesterol (ug/uL) | 0.89 | 0.9 |
| Fat % | Triglyceride (nmol/uL) | 0.48 | 0.81 |
| Fat % | Igf1 mRNA | 0.85 | 0.71 |
| Fat % | Fatty acid (nmol/ul) | 0.97 | 0.19 |
| Fat % | Vanillylmandelic acid (ng/mL) | 0.54 | 0.82 |
| Body length | Leptin (ng/mL) | <0.0001 | 0.35 |
| Body length | Cholesterol (ug/uL) | 0.5 | 0.64 |
| Body length | Triglyceride (nmol/uL) | 0.47 | 0.05 |
| Body length | Igf1 mRNA | 0.88 | 0.20 |
| Body length | Fatty acid (nmol/uL) | 0.47 | 0.68 |
| Body length | Vanillylmandelic acid (ng/mL) | 0.71 | 0.58 |
| Body Weight | Leptin (ng/mL) | <0.0001 | 0.07 |
| Body Weight | Cholesterol (ug/uL) | 0.32 | 0.1 |
| Body Weight | Triglyceride (nmol/uL) | 0.24 | 0.66 |
| Body Weight | Igf1 mRNA | 0.58 | 0.88 |
| Body Weight | Fatty acid (nmol/ul) | 0.19 | 0.62 |
| Body Weight | Vanillylmandelic acid (ng/mL) | 0.15 | 0.88 |

BMC, bone mineral content; BMD, bone mineral density. F-test *p*-values of the direct association between predictor and outcome adjusting for leptin and treatment main effects, and the interaction between treatment and predictor.

| Table S5. Relative gene expression levels were quantified in epididymal fat pad samples using qPCR. | | | | |  |
| --- | --- | --- | --- | --- | --- |
|  | **CS** | **FX** | **IM** | **NR** | **Significance** |
| *Tnf* | 0.881 ± 0.115 | 0.753± 0.087 | 0.75± 0.036 | 1.0 ± 0.087 | *NS* |
| *Slc2a4* | 1.043 ± 0.088 | 0.975 ± 0.098 | 1.04 ± 0.105 | 1.0 ± 0.071 | *NS* |
| *Adipoq* | 1.076 ± 0.055 | 1.018 ± 0.080 | 1.126 ± 0.049 | 1.0 ± 0.033 | *NS* |
| *Pparg* | 1.158 ± 0.093 | 1.078 ± 0.071 | 1.114 ± 0.067 | 1.0 ± 0.057 | *NS* |
| *Lipe* | 1.382 ± 0.112 | 1.546 ± 0.119 | 1.441 ± 0.10 | 1.0 ± 0.034 | *P*=0.002 |
| *Lpl* | 0.959 ± 0.07 | 0.933 ± 0.07 | 0.993 ± 0.040 | 1.0 ± 0.065 | *NS* |
| *Fasn* | 1.149 ± 0.202 | 1.072 ± 0.142 | 1.215 ± 0.123 | 1.0 ± 0.091 | *NS* |
| *Ppargc1a* | 1.014 ± 0.121 | 0.878 ± 0.117 | 1.035 ± 0.130 | 1.0 ± 0.097 | *NS* |

*Tnf*, tumor necrosis factor gene; *Slc2a4*, solute carrier family 2/ facilitated glucose transporter; *Adipoq*, adiponectin gene; *Pparg*, peroxisome proliferator-activated receptor gamma; *Lipe*, lipase E, hormone sensitive type; *Lpl*, lipoprotein lipase; *Fasn*, fatty acid synthase; *Ppargc1a*, peroxisome proliferator activated receptor gamma co-activator 1alpha.
